# Tailoring Tofacitinib Oral Therapy in Rheumatoid Arthritis: The TuTORApp—A Usability Study

**DOI:** 10.3390/ijerph17103469

**Published:** 2020-05-15

**Authors:** Savino Sciascia, Massimo Radin, Irene Cecchi, Pierluigi Di Nunzio, Nicola Buccarano, Federico Di Gregorio, Milone Valeria, Sara Osella, Paola Crosasso, Marika Denise Favuzzi, Elena Rubini, Silvia Grazietta Foddai, Simone Baldovino, Dario Roccatello, Daniela Rossi

**Affiliations:** 1 Center of Research of Immunopathology and Rare Diseases-Coordinating Center of Piemonte and Valle d’Aosta Network for Rare Diseases, and SCDU Nephrology and Dialysis-ERKnet Member, S. Giovanni Bosco Hospital, Department of Clinical and Biological Sciences, 10154 Turin, Italy; massimo.radin@unito.it (M.R.); irene.cecchi@unito.it (I.C.); marika.favuzzi@edu.unito.it (M.D.F.); elena.rubini@unito.it (E.R.); silviagrazietta.foddai@unito.it (S.G.F.); simone.baldovino@unito.it (S.B.); dario.roccatello@unto.it (D.R.); daniela.rossi@unto.it (D.R.); 2 DNDG Srl, 10151 Turin, Italy; gg@dndg.it (P.D.N.); nico@dndg.it (N.B.); fog@dndg.it (F.D.G.); 3 Pharmacy Department, S. Giovanni Bosco Hospital, 10154 Turin, Italy; valeria.milone@aslcittaditorino.it (M.V.); sara.osella@aslcittaditorino.it (S.O.); paola.crosasso@aslcittaditorino.it (P.C.)

**Keywords:** rheumatoid arthritis, mobile application, UX/UI design, gamification

## Abstract

Objective: To create a mobile application able to help patients follow medical treatments properly. Methods: We designed and developed a custom Android/iOS App to remind patients of the pharmaceutical drugs to be taken, of the visits and exams to attend, and to detect their compliance with their personal therapeutic plan. In this paper we describe the App development, UX/UI design, Gamification. TuTOR is an Android and iOS application designed to remind patients of the drugs to be taken, giving them all the information related to their therapeutic plans in a simple and non-invasive way. Thanks to a dedicated back-office, specially designed to meet specific medical information needs, the App can also help physicians detect their patients’ compliance with their treatments and modify prescriptions in real time. The App also ensures a state-of-the-art approach to data security and privacy protection. The main feature of TuTOR is the smart therapy assistant, which features dedicated alarms to remind users of taking their prescription drugs. Thanks to the automatic synchronization with a local database, the alert system works even without connection to the Internet. Particular attention was paid during the App’s design process: we looked to create an intuitive interface to ensure absolute ease of use, with state-of-the-art visual design aimed at maximizing user experience. Other relevant features include the App’s ability to givevisual evidence of the most important drugs to be taken and its note-taking feature, which gives patients the possibility to note down indications on why a specific drug was skipped. The App also keeps track of upcoming medical exams, laboratory tests, and visits on a devoted calendar. It also helps patients by listing therapy contacts, such as physicians’ phone numbers, and indicates all medical references by showing, for example, locations of relevant clinics and pharmacies on a map. Thanks to specific visual progress indicators and an innovative gamification approach, the App encourages users to faithfully follow therapy guidelines. With TuTOR, assessing the therapy’s state of completion is quick and easy.Thanksto the privacy-by-design approach used, all data managed by the system is compliant with the European Privacy Regulation and it is not available to third parties. Expected results: A mobile App for medication adherence might increase objectively and subjectively measured adherence.

## 1. Introduction

Rheumatoid arthritis (RA) is one of the most common autoimmune diseases, mainly characterized by chronic inflammation and persistent synovitis, which can lead to joint destruction and permanent damage. The introduction of biological agents and, more recently, small oral molecules has changed the scenario of pharmacologic treatment of RA and it has become a real revolution. These drugs have innovative mechanisms of action, based on the inhibition of specific molecular or cellular targets directly involved in disease pathogenesis [1,2,3]. 

Among small oral molecules, tofacitinib is an oral Janus kinase inhibitor approved for the treatment of RA either as a monotherapy or in combination with conventional synthetic disease modifying anti-rheumatic drug (csDMARDs). The efficacy and safety of tofacitinib 5 mg and 10 mg twice daily administered as monotherapy or in combination with csDMARDs (mainly methotrexate) in patients with active RA, have been demonstrated in two phase three studies [4,5]. One of most interesting peculiarities of tofacitinib, when compared to the majority of the biological drugs used for the treatment of RA, is the oral administration. However, while oral administration is undoubtedly considered an advantage from a patients’ perspective, adherence to self-management and medication might raise some concerns in treating physicians. This is particularly true in the management of chronic conditions such as RA. 

Notably, shifting medications, poly-pharmacy, and complexities of daily life are likely to contribute to patients’ inability to deal adequately with their medical conditions, potentially leading to poor disease control and/or management of co-morbidities. In RA, in particular, it is well known that cardiovascular (CV) disease represents the most frequent cause of morbidity and mortality, accounting for 39%–50% of deaths in this particular subgroup of subjects [6]. Nonetheless, up to 50% of RA patients stop taking medication for CV comorbidities during the first year of prescription [7,8,9,10]. Besides, a proper adherence to self-management and medication can be even more challenging in specific sub-populations of RA patients, such as the elderly because of the concurrent use of multiple medications and reduced awareness of their medical condition. 

Novel strategies are warranted to better monitor and address the needs of chronically ill patients that are self-managing their often complex treatment. In this context, mobile information technology may offer new solutions to better meet these needs. In fact, these technologieshave already been applied for other diseases and has demonstrated to substantially improve pharmaceutical adherence [11,12].

We aim to develop a mobile application to support the management of RA patients treated with tofacitinib in order to improve their therapy adherence: the TuTOR (Tailoring Tofacitinib Oral therapy in Rheumatoid arthritis) mobile App. The project is divided in two main phases. The first phase (Phase I) includes the TuTOR mobile App design and development. A second phase (Phase II) is based on the TuTOR mobile App clinical validation. In phase II the effect on participants’ reported adherence to medication, laboratory parameters, and clinical follow-up will be evaluated. Adherence to therapy will be investigated through objective information acquired from the logged interaction protocols and users’ subjective assessments and quality of life questionnaires. In addition, the influence on affinity for technology will be evaluated at baseline in order to determine if particular subgroups of RA patients will benefit more from the use of the mobile App.

Herewith, we aim to describe the study protocol and the milestones of Phase I of the TuTOR project which led to the App development. 

## 2. Methods

### 2.1. Phase I: TuTOR Mobile App Design and Development 

We designed and developed a custom Android/iOS App to remind patients of the pharmaceutical drugs to be taken, of the visits and exams to attend, and to detect their compliance with their personal therapeutic plan. All of the materials will be available in English and Italian (the native language of the participants). More information on the technical aspects of the TuTOR developing architecture is provided as Supplementary Material. 

Taking medication accurately means better therapy results. Drug therapies with complex intake timetables are difficult for patients to follow and measuring the medical effectiveness of a therapy with a discontinuous or inaccurate application can lead to unreliable results: this is why the TuTOR App idea took shape. TuTOR is an Android and iOS application designed to remind patients of the drugs to be taken, giving them all the information related to their therapeutic plans in a simple and non-invasive way. The App also ensures a *state-of-the-art* approach to data security and privacy protection.

#### 2.1.1. Not Just A Reminder: Smart Therapy Assistant

The main feature of TuTOR is the smart therapy assistant, which features dedicated alarms to remind users of taking their prescription drugs. Thanks to the automatic synchronization with a local database, the alert system works even without internet access. This ensures that alerts work in all conditions, a crucial element to help patients never forget their medications. Particular attention was paid during the App’s design process, particularly focusedto create an intuitive interface to ensure absolute ease of use, with state-of-the-art visual design aimed at maximizing user experience. Other relevant features include the App’s ability to give visual evidence of the most important drugs to be taken and its note-taking feature, which gives patients the possibility to note down indications on why a specific drug was skipped. The App also keeps track of upcoming medical exams, laboratory tests, and visits on a dedicated calendar. It also helps patients by listing therapy contacts, such as physicians’ phone numbers, and indicates all medical references by showing, for example, locations of relevant clinics and pharmacies on a map. Thanks to specific visual progress indicators and an innovative gamification approach, the App encourages users to faithfully follow therapy guidelines. With TuTOR, assessing the therapy’s state of completion is quick and easy.

#### 2.1.2. Medical Plan

In this form, the patient will find all listed medications that they are currently taking. Information provided will include the name of the medication, the number of intakes per day, and corresponding doses. The patient will not be able to change the list of medications, only the investigator of the study will be provided with the code to change the “Medical Plan” settings. The patients will have to check the boxes each day when taking every medication suggested by the TuTOR mobile App with the possibility to recover a wrong information if given a wrong input. 

**Figure 1:**
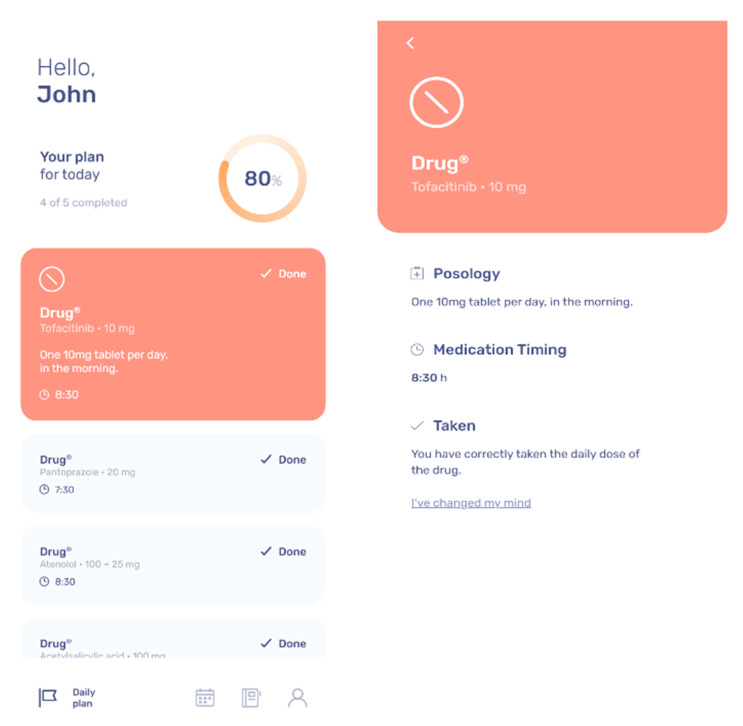
Medical plan in the TuTOR mobile App.

Figure 1 shows the medical plan in the TuTOR mobile App.

#### 2.1.3. Clinical Follow-up

**Figure 2:**
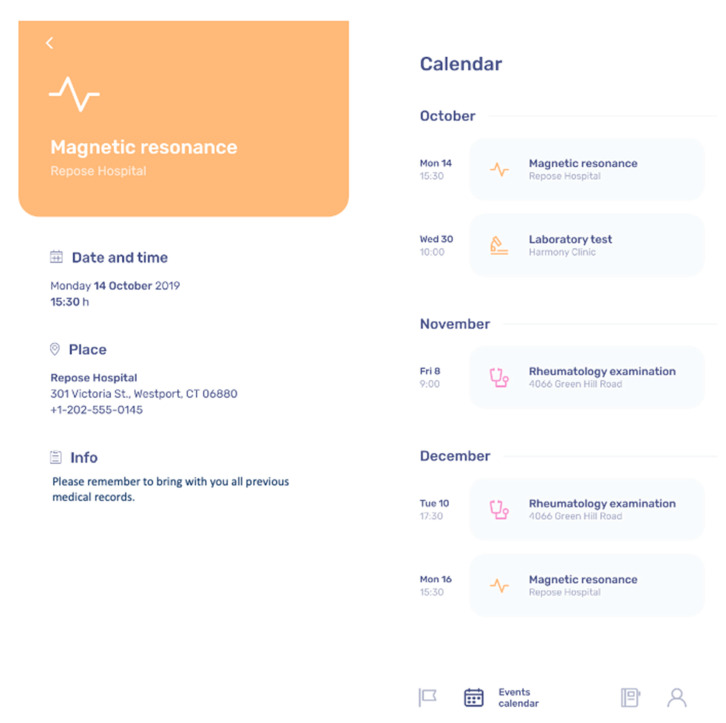
Clinical follow-up plan in the TuTOR mobile App.

In this form, the patient will find all planned follow-up clinical appointments according to the treating clinician indications. Information provided will include follow-up visits (date, time, location, and name of the reference Doctor of the center), specialist appointments (date, time, location, and name of the Specialist Doctor), instrumental testing (date, time, and location), and laboratory testing (date, time, and location). The patient will not be able to change the planned follow-up list, only the investigator of the study will be provided with the code to change the “Clinical Follow-up” settings. The patients will have to check the boxes for each follow-up visit performed suggested by the TuTOR mobile App with the possibility to recover a wrong information if given a wrong input. Figure 2 shows the clinical follow-up plan in the TuTOR mobile App.

### 2.2. Phase II: Validation of the TuTOR App

#### 2.2.1. Clinical Validation of TuTOR Mobile App Protocol 

This study will include a total of 20 RA patients that will begin treatment with tofacitinib jointly to the use of the TuTOR mobile App, developed to improve adherence to self-management, clinical follow-up, and medication monitoring. Patients will be followed-up for 6 months, with personal questionnaires at baseline and every 3 months. Clinical and laboratory follow-up will be evaluated by the treating clinician according to patient’s comorbidities and specific needs. 

At baseline, with the help of certified questionnaires (drafted in the native language of the participants), technologyliteracy and digital health literacy will be evaluated in order to assess the affinity for technology and its influence on the outcomes of the study. Adherence to therapy will be investigated from one hand through objective information acquired from the logged interaction protocols. From the other hand, this study will use certified quality of life questionnaires and adherence to therapy questionnaires in order to assess qualitative indicators and subjective feedbacks from the patients and their experience in using the TuTOR App. 

The recruited patients will then receive a personal learning-by-doing tutorial session and introduction to the TuTOR mobile App, installed on a tablet or smartphone. The participants will be familiarized with the functions of the application (i.e., confirming medication intake and recording follow-up visits and clinical appointments) and how to recover if a wrong input had been made. 

**Figure 3:**
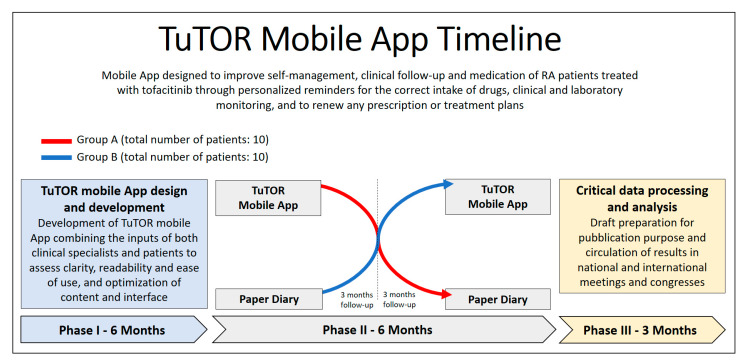
Timeline of the research protocol of the study.

The study will be conducted using a crossover design with three sequences: initial phase (training and baseline assessment), interventional phase (3 months of therapy using the App system), and comparative phase (3 months of treatment using a paper-diary). Users will experience the interventional and comparative phases alternately. Figure 3 shows the timeline of the research protocol of the study.

#### 2.2.2. Patients Characteristics 

##### Inclusion Criteria

Individuals aged 18 years or older who meet the 2010 ACR and EULAR classification criteria for RA [13] with active diseasedefined as having four or more tender or painful joints on motion and four or more swollen joints (based on a 28 joint count) at baseline despite treatment with methotrexate (15–25 mg per week), high-sensitivity C-reactive protein of ≥3 mg/L, and class I–III functional capacity as classified by the ACR 1991 revised criteria for global functioning status in RA [14]. Patients are required to discontinue all csDMARDs, other than methotrexate, for at least 4 weeks or five half-lives, whichever was longer, before baseline, but could continue to receive stable non-steroidal anti-inflammatory drugs, analgesics, or oral corticosteroids, or a combination, throughout the study. Patients who had responded inadequately or had an adverse event secondary to treatment with a biological DMARD (bDMARD) could be included after having discontinued the bDMARD for a minimum period of time depending on the specific agent (i.e., rituximab or other selective B lymphocyte depleting agents 52 weeks; abatacept, certolizumab pegol, and tocilizumab 12 weeks; golimumab 10 weeks; infliximab 8 weeks; and anakinra and etanercept 4 weeks).

##### Exclusion Criteria

Contraindications for any study treatment; a history of infections requiring treatment within 2 weeks, or any admission to hospital within the 6 months before the beginning of the study; had exclusionary morbidities, HIV, hepatitis B or C; inadequately treated or undocumented treatment for tuberculosis; one episode of disseminated herpes zoster or herpes simplex; any clinically significant laboratory abnormalities; ongoing pregnancy. Exclusion criteria include also patients who had previously received tofacitinib, or live attenuated vaccines other than the herpes zoster vaccine (within 6 weeks before study initiationor planned within 6 weeks after discontinuation of study treatment). 

### 2.3. Data Analysis

#### 2.3.1. Objective Indicators for Therapy Adherence and Clinical Follow-up

Confirmation rates of medication intake and the number of follow-up records in mobile App and in the paper-diary will be analyzed. More in detail, the patient will have the option to choose why they are not taking the medication: (a) forgetfulness, (b) intolerance/allergy reaction, (c) scarce efficacy. In order to be able to compare the outcomes of the interventions, a target–performance comparisonwill be performed. For medication, the target is defined as the number of medications each participant has to take each day multiplied by the days they actually used the system or the diary. The variable “absolute performance” is defined as the actual number of confirmations made by the patient that they took the medication. Having to take >1 unit (i.e., >1 pill) of a single medication at the same point in time or on the same day is considered as one medication intake (“all or none”) as the application cannot register whether participants only took a subset of a particular medication. The rate of adherence to confirm medication then will be the ratio of performance and target multiplied by 100 to obtain a percentage value. Assessing adherence for clinical follow-up recording isperformed similarly, as participants have a tailored follow-up depending on the single comorbidities and different follow-up visits. Every specialist visit is considered as one follow-up confirmation, as well as every laboratory monitoring and instrumental tests. Similarly, to medication, the rate of adherence to clinical follow-up will be the ratio of performance and target multiplied by 100 to obtain a percentage value.

#### 2.3.2. Back-Office

**Figure 4:**
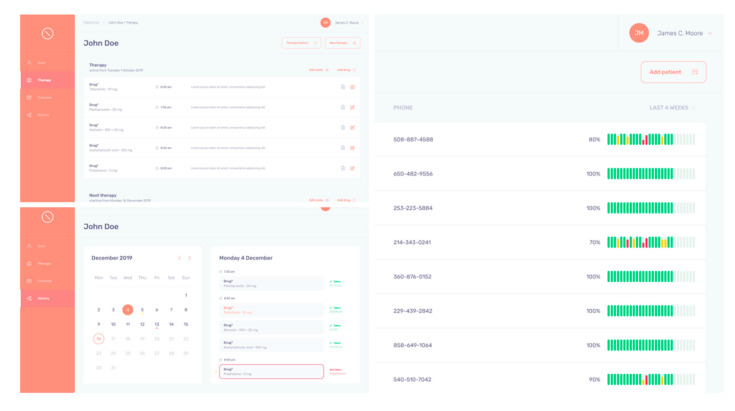
Back-office of the TuTOR App.

The TuTOR App features a complete dashboard to allow physicians to keep their patients’ therapies under control, monitoring their adherence toprescriptions (Figure 4). In TuTOR, physicians also have all patients’ relevant medical information under control. The back-office is designed to keeptrack, visit after visit, of drugs, dosages, times of intake, and previous therapies. All data is synchronized in real time on patients’smartphones, giving physicians the possibility to modify therapies in a timely manner. Thanks to the privacy-by-design approach used, all data managed by the system is compliant with the European Privacy Regulation and it is not available to third parties.

#### 2.3.3. Subjective Indicators for Therapy Adherence and Clinical Follow-up

Technical knowledge and experience are assessed using an adapted version of the computer literacy scale (CLS) [15]. Subjective adherence are determined by the A14-scale, which contains 14 items that ask participants to report on a five-point scale, ranging from “never” (0) to “very often” (4), their behavior with respect to medication adherence and the degree to which various barriers to adherence apply to them [16]. Based on the A14-scale, values < 50 are regarded as non-adherent and values between 50 and 56 as adherent (sums ranging between 0 and 56). 

A questionnaire investigating quality of life and adherence to therapy (supplement A) will also be implemented in the follow-up visits to determine the influence of the TuTOR App in the aspects of daily life and overall patients’ satisfaction. 

#### 2.3.4. Statistical Analysis Plan

Data will be analyzed using SPSS statistics software version SPSS 26.0 (IBM, Armonk, New York). A multifactorial analysis of variance (ANOVA) with repetitions for the different factor levels of the response variables with a significance level of 0.05 was will be conducted. Significant findings will be additionally analyzed by post hoc analysis and Bonferroni correction to minimize the Type-I error rate because of multiple paired comparisons of mean values. Multivariate logistic regression analysis will be performed to identify significant independent factors.

## 3. Expected Results and Conclusions

We anticipate that the mean for subjectively assessed adherence will be more pronounced after the interventional phase than after the comparative phase. Similarly, we expect a better adherence to be associated to an adequate control of disease activity and amelioration of inflammatory laboratory parameters. 

A mobile App for medication adherence might increase objectively and subjectively measured adherence in 20 RA patients treated with tofacitinib. The findings might have promising clinical implications: digital tools can assist chronic disease patients achieving better adherence to medication, leading to an improved disease management. Ideally, although this requires initial offline training, it can reduce complications and clinical overload because of non-adherence.

## Supplementary Materials

Addendum 1: App development: Technical Aspects.

## References

[1] Wiens A., Venson R., Correr C.J., Otuki M.F., Pontarolo R. (2010). Meta-analysis of the efficacy and safety of adalimumab, etanercept, and infliximab for the treatment of rheumatoid arthritis. Pharmacotherapy.

[2] Hahn B.H., McMahon M.A., Wilkinson A., Wallace W.D., Daikh D.I., Fitzgerald J.D., Karpouzas G.A., Merrill J.T., Wallace D.J., Yazdany J. (2012). American College of Rheumatology guidelines for screening, treatment, and management of lupus nephritis. Arthritis Care Res. (Hoboken).

[3] Smolen J.S., Landew R., Bijlsma J., Burmester G., Chatzidionysiou K., Dougados M., Nam J., Ramiro S., Voshaar M., van Vollenhoven R. (2017). EULAR recommendations for the management of rheumatoid arthritis with synthetic and biological disease-modifying antirheumatic drugs: 2016 update. Ann. Rheum. Dis..

[4] Burmester G.R., Blanco R., Charles-Schoeman C., Wollenhaupt J., Zerbini C., Benda B., Gruben D., Wallenstein G., Krishnaswami S., Zwillich S.H. (2013). Tofacitinib (CP-690,550) in combination with methotrexate in patients with active rheumatoid arthritis with an inadequate response to tumour necrosis factor inhibitors: A randomised phase 3 trial. Lancet.

[5] Fleischmann R., Kremer J., Cush J., Schulze-Koops H., Connell C.A., Bradley J.D., Gruben J.D., Wallenstein G.V., Zwillich S.H. (2012). Placebo-Controlled Trial of TofacitinibMonotherapy in Rheumatoid Arthritis. N. Engl. J. Med..

[6] Del Rincón I.D., Williams K., Stern M.P., Freeman G.L., Escalante A. (2001). High incidence of cardiovascular events in a rheumatoid arthritis cohort not explained by traditional cardiac risk factors. Arthritis Rheum..

[7] Vrijens B., Vincze G., Kristanto P., Urquhart J., Burnier M. (2008). Adherence to prescribed antihypertensive drug treatments: Longitudinal study of electronically compiled dosing histories. BMJ.

[8] Melnikow J., Kiefe C. (1994). Patient compliance and medical research: Issues in methodology. J. Gen. Intern. Med..

[9] Cramer J.A., Benedict A., Muszbek N., Keskinaslan A., Khan Z.M. (2008). The significance of compliance and persistence in the treatment of diabetes, hypertension and dyslipidaemia: A review. Int. J. Clin. Pract..

[10] Hamood H., Hamood R., Green M.S., Among R. (2016). Determinants of adherence to evidence-based therapy after acute myocardial infarction. Eur. J. Prev. Cardiol..

[11] Grindrod K.A., Li M., Gates A. (2014). Evaluating user perceptions of mobile medication management applications with older adults: A usability study. JMIR mHealth uHealth.

[12] Mertens A., Brandl C., Miron-Shatz T., Schlick C., Neumann T., Kribben A., Meister S., Diamantidis C.J., Albrecht U.-V., Horn P. (2016). A mobile application improves therapy-adherence rates in elderly patients undergoing rehabilitation. Medicine.

[13] Aletaha D., Neogi T., Silman A.J., Funovits J., Felson D.T., Bingham C.O., Birnbaum N.S., Burmester G.R., Bykerk V.P., Cohen M.D. (2010). 2010 Rheumatoid arthritis classification criteria: An American College of Rheumatology/European League Against Rheumatism collaborative initiative. Arthritis Rheum..

[14] Hochberg M.C., Chang R.W., Dwosh I., Lindsey S., Pincus T., Wolfe F. (1992). The American College of Rheumatology 1991 revised criteria for the classification of global functional status in rheumatoid arthritis. Arthritis Rheum..

[15] Steele R., Lo A., Secombe C., Wong Y.K. (2009). Elderly persons’ perception and acceptance of using wireless sensor networks to assist healthcare. Int. J. Med. Inform..

[16] Jank S., Bertsche T., Schellberg D., Herzog W., Haefeli W.E. (2009). The A14-scale: Development and evaluation of a questionnaire for assessment of adherence and individual barriers. Pharm. World Sci..

