# Supplementary material for: Tailoring Tofacitinib Oral Therapy in Rheumatoid Arthritis: The TuTORApp—A Usability Study
Source: Int J Environ Res Public Health. 2020 May 15;17(10):3469. doi: 10.3390/ijerph17103469 (PMC7277549; doi:10.3390/ijerph17103469)
Supplement: Supplementary file 1 [file ijerph-17-03469-s001.pdf]

TuTOR App is a cloud native system composed of distributed microservices in order to be resilient and scalable avoiding single point of failure.

It is based on a full stack opensource framework, and state-of-the-art privacy by design patterns.

The core back-end is based on a dotnet core 2.2 [1] environment with a GRAPH-QL [2] endpoint that interacts with a PostgreSQl [3] RDBMS. The authentication is operated using JWT [4] tokens and OAuth 2 [5] standard protocol features.

The Android and iOS application is a hybrid Xamarin/VueJs [6,7] client connected to a SQLite [8] database operating on the smartphone that is synchronized in realtime using SignalR [9] through an HTTP/S websocket channel. This architecture ensures that all the main features and in particular alarms can work even without an internet connection. This is a crucial point to avoid missing alerts on therapies and drugs to be taken and to allow collecting all user interaction data that can be used to better the quality and the usability of the application.

The physician dashboard is a Vuejs application written in typescript, connected to the backend via a GRAPH-QL client. The study on personas helped us to give complete control to the physician over the patients' therapies in order to help to verify the overall compliance at glance.

[1] DotNet Core 2.2 https://dotnet.microsoft.com/learn/dotnet/what-is-dotnet
[2] GRAPH-QL https://graphql.org/
[3] PostgreSQL https://www.postgresql.org/
[4] JWT https://jwt.io/
[5] OAuth 2 https://tools.ietf.org/html/rfc6749
[6] Xamarin https://dotnet.microsoft.com/apps/xamarin
[7] VueJs https://vuejs.org/
[8] SQLite https://www.sqlite.org/about.html
[9] SignalR https://dotnet.microsoft.com/apps/aspnet/signalr
